# Deciphering the pharmacological mechanisms of *Chaenomeles Fructus* against rheumatoid arthritis by integrating network pharmacology and experimental validation

**DOI:** 10.1002/fsn3.2938

**Published:** 2022-07-07

**Authors:** Mengjia Sun, Haijun Zhao, Yuecheng Liu, Yanni Ma, Zhenhua Tian, Huanjun Wang, Sheng Wei, Qingmei Guo, Zhengwei Gu, Haiqiang Jiang

**Affiliations:** ^1^ School of Pharmaceutical Sciences Shandong University of Traditional Chinese Medicine Jinan China; ^2^ School of Traditional Chinese Medicine Shandong University of Traditional Chinese Medicine Jinan China; ^3^ Shandong Academy of Traditional Chinese Medicine Jinan China; ^4^ Experiment Center, Shandong University of Traditional Chinese Medicine Jinan China; ^5^ Key Laboratory of Traditional Chinese Medicine Classical Theory, Ministry of Education Shandong University of Traditional Chinese Medicine Jinan China; ^6^ Shandong Provincial Key Laboratory of Traditional Chinese Medicine for Basic Research Shandong University of Traditional Chinese Medicine Jinan China

**Keywords:** Chaenomeles Fructus, experimental validation, mechanism, network pharmacology, rheumatoid arthritis

## Abstract

*Chaenomeles Fructus* is a plant that can be used for both food and medicine. Modern studies have shown that *Chaenomeles Fructus* has anti‐inflammatory and immunosuppressive effects on arthritis. However, the mechanism of action of *Chaenomeles Fructus* on rheumatoid arthritis (RA) and its main active ingredients are still unclear. This study was aimed at devising an integrated strategy for investigating the bioactivity constituents and possible pharmacological mechanisms of *Chaenomeles Fructus* against RA. The components of *Chaenomeles Fructus* were analyzed using UPLC‐Q‐Exactive orbitrap MS techniques and applied to screen the active components of *Chaenomeles Fructus* according to their oral bioavailability and drug‐likeness index. Then, we speculated on the potential molecular mechanisms of *Chaenomeles Fructus* against RA through a network pharmacology analysis. Finally, the potential molecular mechanisms of *Chaenomeles Fructus* against RA were validated in a complete Freund's adjuvant (CFA)‐induced RA rat model. We identified 48 components in *Chaenomeles Fructus* and screened seven bioactive ingredients. The results of the network pharmacology prediction and the experimental verification results were analyzed by Venn analysis, and the experimental results concluded that *Chaenomeles Fructus* mainly interferes with the inflammation of RA by inhibiting arachidonic acid metabolism and the MAPK signaling pathway. This study identified the ingredients of *Chaenomeles Fructus* by UPLC‐Q‐Exactive orbitrap MS and explained the possible mechanisms of *Chaenomeles Fructus* against RA by integrating network pharmacology and experimental validation.

## Graphical abstract

Taking all the above results together, we identified 48 chemical components from Chaenomeles Fructus based on UPLC‐Q‐Exactive orbitrap MS, which mainly includes four types: amino acids, organic acids, flavonoids, and triterpenes. Moreover, we predicted the mechanism of action of Chaenomeles Fructus in the treatment of RA with network pharmacology and verified it through CFA‐induced RA in rats. The results of the network pharmacology prediction and the verification experimental results were analysed by Venn analysis, and the experimental results concluded that Chaenomeles Fructus mainly interferes with the inflammation of RA by inhibiting arachidonic acid metabolism and the MAPK signalling pathway.

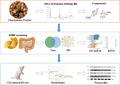

## 1 INTRODUCTION

*Chaenomeles Fructus*, the dry and near mature fruit of the Rosaceae plant *Chaenomeles speciosa* (Sweet) Nakai, dispels wind and dehumidification and can be used to prevent and treat rheumatism, cholera, dysentery, enteritis, beriberi, vitamin C deficiency, etc. (Hou, 2011; Hu et al., 2021; Jiangsu New Medical College, 1997; Qin et al., 2015; Zhang et al., 2018). Modern studies have shown that *Chaenomeles Fructus* has anti‐inflammatory and immunosuppressive effects on arthritis (Gao, 2007; Hou, 2011; Hu et al., 2021; Zhang et al., 2018). Some of the main constituents in *Chaenomeles Fructus*, including oleanolic acid, betulinic acid, and ursolic acid, possess potential anti‐inflammatory properties (Zhang et al., 2014). The anti‐inflammatory effect of *Chaenomeles Frctus* is the holistic effect of the combination of its multiple components. However, the mechanism of action of *Chaenomeles Fructus* on RA and the main active ingredients are still unclear.

Rheumatoid arthritis (RA) is a chronic autoimmune joint disease characterized by inflammation of synovial tissue, which can cause cartilage and bone damage in addition to disability (Smolen et al., 2016). It affects approximately 1% of the population worldwide, and its current treatment strategies are costly (Silman & Pearson, 2002). Moreover, in such disorders, inflammation can further extend to damage other body organs than the joints, comprising the heart, lungs, eyes, and skin (Cojocaru et al., 2010). Currently, medications for the treatment of RA, such as nonsteroidal anti‐inflammatory drugs (NSAIDs) and disease‐modifying antirheumatic drugs (DMARDs), have serious side effects, including cardiovascular diseases and hepatotoxicity, which limit their extensive clinical use (Chen et al., 2020). Given the side effects of existing therapies, such as limited efficacy, potential toxicity, and high cost, many countries have paid great attention to herbal therapy (Kuwana et al., 2018). For example, Tripterygium glycosides have anti‐inflammatory and immunomodulatory effects and are considered to be the most effective medicinal plants for treating RA in China. However, the side effects and toxicity of Tripterygium glycosides cannot be ignored. Thus, it is necessary to find drugs with good curative effects and few side effects.

With the rise of systems biology, network pharmacology uses big data to visualize the connections of complex systems and to provide new ideas and approaches for the study of mechanisms in treating diseases (Wang et al., 2021). It uses a variety of analytical tools to extract relevant data from massive amounts of biological information and medicinal plant information to build disease gene or medicinal plant active ingredient–protein target interaction networks for data mining, thereby establishing the disease regulatory networks of medicinal plants and their formulas. The synergistic mechanisms of the complicated medicinal plant formulas can be elucidated in greater depth by combining the results from proteomics, transcriptomics, or metabolomics. For instance, in a recent study, the mechanisms of Shenyan Kangfu tablets in treating diabetic nephropathy were studied through network pharmacology combined with metabolomics (Wang et al., 2021).

**FIGURE 1:**
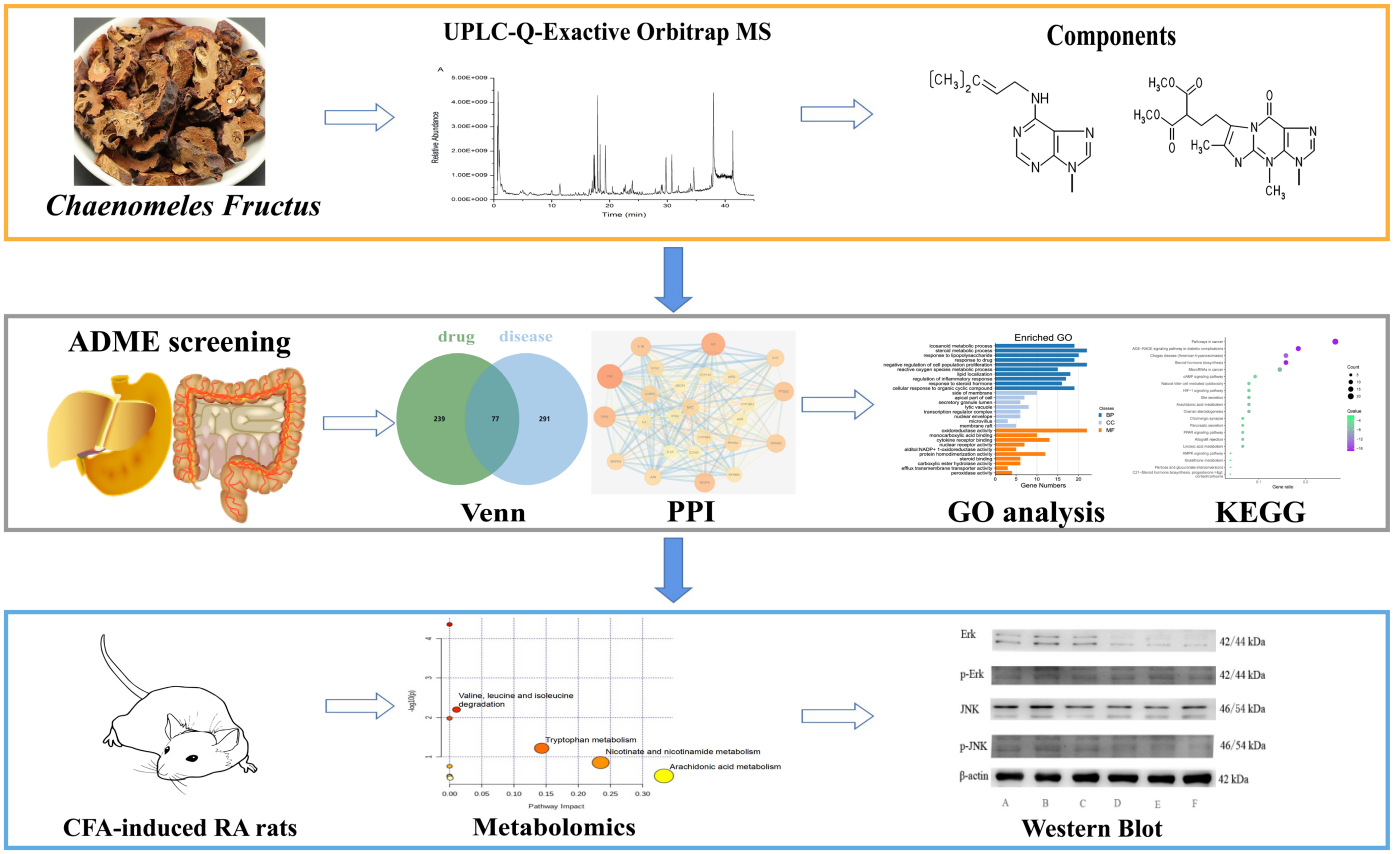
Flowchart of the whole study including all the groups. The first level (orange box) is the identification process of *Chaenomeles Fructus* components; the second level (gray box) is the network pharmacology prediction process; the third level (blue box) is the experimental verification part, including metabolomics and related protein detection

This study, based on the scientific strategy of network pharmacology, aimed to systematically investigate the predicted therapeutic targets and biological signaling pathways of *Chaenomeles Fructus* against RA. In addition, we established a complete Freund's adjuvant (CFA)‐induced RA rat model for verification. A metabolomics method based on UPLC‐Q‐Exactive orbitrap MS was used to collect the serum metabolic profiles of rats and explore the metabolic changes that occurred after *Chaenomeles Fructus* treatment. The MAPK signaling pathway involved in MAPK3 targets was selected for validation in the current models. The specific experimental process is shown in Figure 1.

## 2 MATERIALS AND METHODS

### 2.1 Chemicals and reagents

The reference standards for oleanolic acid, betulinic acid, maslinic acid, quercetin, rutin, quercetin, hyperin, protocatechuic acid, chlorogenic acid, shikimic acid, gallic acid, caffeic acid, kojic acid, cinnamic acid, vanillic acid, benzoic acid, p‐hydroxybenzoic acid, succinic acid, fumaric acid, and L‐3‐phenyl lactic acid were purchased from Minster Co., Ltd. (Chengdu, China). Acetonitrile, methanol, and formic acid (HPLC grade) were obtained from Fisher Company, USA. 10% chloral hydrate was purchased from Damao Chemical Reagent Factory Ltd. (Tianjin, China). Complete Freund's adjuvant (CFA) was obtained from Sigma Aldrich (Milan, Italy); Tripterygium Glycosides (TG) were purchased from Meitong Pharmaceutical Co., Ltd. (Jiangsu, China).

*Chaenomeles Fructus* was obtained from Anhui People's Traditional Chinese Medicine Pieces Co., Ltd. (Anhui, China) and identified by Prof. Qingmei Guo, Shandong University of Traditional Chinese Medicine.

### 2.2 UPLC‐Q‐Exactive orbitrap MS for chemical characterization

#### 2.2.1 UPLC‐Q‐Exactive orbitrap MS conditions

UPLC‐Q‐Exactive orbitrap MS analysis was used for a comprehensive analysis of the constituents in *Chaenomeles Fructus* extract. The chromatography system was equipped with an autosampler, a diode‐array detector, a column compartment, and two pumps. The chromatographic conditions were optimized, and a Halo C18 column (2.1 × 100 mm, 2.7 μm, AMT, USA) maintained at 40°C was finally chosen for separation of *Chaenomeles Fructus* alcohol extract. The mobile phase consisted of solvent A (0.05% formic acid in water) and solvent B (0.05% formic acid in acetonitrile). The following gradient program was used: 0–5 min 5% B, 5–15 min 5% B to 15% B, 15–17 min 15% B to 45% B, 17–40 min 45% B to 95% B, 40–40.1 min 95% B to 5% B, 40.1–45 min 5% B. The flow rate was set at 0.3 ml/min. The injection volume was 3.0 μl, and the sampler was set at 4°C.

Meanwhile, mass spectrometry detections were set as follows: capillary temperature 350°C; S‐lens RF level 55.0; spray voltage 3.5 kV; sheath gas (N_2_) flow rate 45 arb; auxiliary gas (N_2_) flow rate 15 arb; mass spectrometry acquisition range 80–1200 *m/z*; and resolution 17,500. Data acquisition is done through Xcalibur 4.1 software operated in positive and negative ion mode.

#### 2.2.2 Sample preparation

The *Chaenomeles Fructus* was ground into powder, accurately weighed 0.5 g, dissolved in methanol with sonication for 30 min, and then use methanol to supplement the weight loss. The solution was further filtrated through 0.22 μm membrane for LC–MS/MS analysis.

#### 2.2.3 Preparation of standard solution

Precisely weigh 2.00 mg of the above‐mentioned standard product and place it in a 10 ml volumetric flask. Dissolve to a fixed volume with methanol as the standard solution and store it at 4°C. The mixed solution of the standard was diluted 100 times with methanol and treated with ultrasound at room temperature for 30 min. Methanol was added to make up the weight loss and filtered through 0.22 μm membrane.

### 2.3 Network pharmacology study

The construction of the network was mainly based on the overall prediction of the TCMSP (http://ibts.hkbu.edu.hk/lsp/tcmsp.php), Swiss TargetPrediction (http://www.swisstargetprediction.ch/), TTD (https://db.idrblab.org/ttd/), OMIM (http://omim.org/), STRING (https://string‐db.org/), and Metascape (http://metascape.org/) databases for the treatment of RA with *Chaenomeles Fructus*. The procedure for network construction was as follows: (1) Based on the qualitative identification results of mass spectrometry, compounds that met the requirements of oral bioavailability (OB)  ≥  30% and drug‐likeness (DL)  ≥  0.18 were extracted from the TCMSP database, and the potential active compounds were screened out. Their corresponding targets were queried in the TCMSP and Swiss TargetPrediction databases (Daina et al., 2019; Ru et al., 2014). (2) We collected gene targets for RA from two sources. The first source was the TTD (http://db.idrblab.net/ttd/) (Wang, Yu, et al., 2020). We used the keyword “rheumatoid arthritis” to search this database. The second source was the Online Mendelian Inheritance in Man (OMIM) database (www.omim.org/, updated on 28 February 2019) (Hamosh et al., 2005). (3) First, we intersected the obtained drug targets with the genes associated with disease and obtained a Venn diagram of the intersected gene symbols. These overlapping targets were further checked and retrieved into UniProt ID by using UniProt (https://www.uniprot.org/) (The UniProt Consortium, 2018). (4) The protein–protein interaction (PPI) analysis was performed by employing String (https://string‐db.org/) (Szklarczyk et al., 2019) and visualized by Cytoscape 3.8.2 (Shannon, 2003). The action targets of *Chaenomeles Fructus* on RA ulcers were uploaded from the Metascape (http://metascape.org/) database, and the functions of biological process (BP), cellular component (CC), and molecular function (MF) were obtained by enrichment, and an enrichment analysis was carried out (Zhou, Yu, et al., 2019). Through the analysis of KEGG signaling pathways in the Metascape database, we comprehensively predicted the biological characteristics and regulatory pathways of *Chaenomeles Fructus* acting on RA targets. The calculation formula (RichFather  =  the number of genes belonging to this pathway in the target gene set/the number of all genes in this pathway in the background gene set) was adopted, and the bubble chart was drawn (Li et al., 2020).

### 2.4 Experimental validation

#### 2.4.1 Animals

Male Sprague–Dawley (SD) rats (weight, 160–200 g) were purchased from Beijing Weitong Lihua Experimental Animal Technology Co., Ltd (Beijing, China; Certificate No. SCXK 2016–0006). All rats were housed in a specific pathogen‐free (SPF) facility at a constant temperature of 23° ± 1°C with a relative humidity environment of 55% ± 5% and a standard 12 h/12 h (light/dark) cycle. Animals were allowed free access to water and fed a unified basic diet. Prior to the start of the experiment, the animals were maintained in hygienic conditions for at least a week to adapt to the environment. All animal welfare and experimental procedures were performed in accordance with the National Institutes of Health Guide for the Care and Use of Laboratory Animals, and the protocols used were approved by the Animal Ethics Committee of Shandong University of Traditional Chinese Medicine Laboratory Animal Center, Jinan, China.

#### 2.4.2 Animal experiments

Animals were randomly divided into six groups (11 rats/group). Each rat was injected with 0.1  ml CFA (10  mg/ml) both in and around the articular cavity, except for the control group. Treatment of rats began 1  day after induction. Group 1 included nonimmunized rats (control), and rats in Groups 2–6 included animals receiving the experimental drug. Group 2 included rats treated with intragastric saline administration (model). Group 3 included rats treated with intragastric *Chaenomeles Fructus* at 0.15  g/kg/day (low). Group 4 included rats treated with intragastric *Chaenomeles Fructus at* 0.30  g/kg/day (medium). Group 5 included rats treated with intragastric *Chaenomeles Fructus at* 0.60  g/kg/day (high). Group 6 included rats treated with intragastric TG at 0.009  g/kg/day (TG). Rats received administration for 3  weeks. After 24  h of the last administration, all animals were anesthetized with 1.5% pentobarbital sodium, and blood samples and synovial tissue were collected. Blood samples were drawn into the Eppendorf tubes, allowed to clot for 30  min, and then centrifuged (999 *g*, 4°C) for 15  min to obtain serum samples. The serum samples and synovial tissue were stored at −80°C until analysis.

#### 2.4.3 Measurement of paw swelling

Paw swelling was evaluated by measuring the diameters (mean of three readings) of both the injected and noninjected paws using a toe volume measuring instrument (Calvin Biotechnology Co., Ltd., China) before (Day 0) and after (hour 4, Day 7, Day 21) CFA immunization. The difference in the paw volumes after and before inflammation at certain time points indicated the paw swelling degree of rats at that time point (mL  =  volume of inflamed paw volume of noninflamed paw) (Zhou, Zhou, et al., 2019).

#### 2.4.4 Serum untargeted analysis by UPLC‐Q‐Exactive orbitrap MS

##### Preparation of serum samples

When the serum metabolites were analyzed, the serum samples were melted at 4°C. Serum samples (100  μl) and acetonitrile (400  μl) were mixed in a tube to remove proteins from the serum, including 2‐chloro‐L‐phenylalanine (0.05  mg/ml, 15  μl) as an internal standard. The mixture was vortexed for 2  min, allowed to stand at 4°C for 10  min, and then centrifuged at 15,984 *g* for 20  min at 4°C. The supernatant (400  μl) was placed in a 2  ml EP tube, dried with nitrogen, and then redissolved by adding the initial mobile phase of 100  μl. The solution was centrifuged at 12,000  rpm for 5  min at 4°C, and 70  μl of the supernatant was injected into the column for LC–MS analysis.

##### LC–MS/MS conditions

Ultra‐performance liquid chromatography–electrospray ionization tandem mass spectrometry (UPLC‐ESI‐MS) analysis was performed using a hybrid Quadrupole‐Orbitrap high‐resolution mass spectrometer (Q‐Exactive, QE) coupled with an Ultimate 3000 UPLC system (Thermo Fisher, USA). Data acquisition was performed by Xcalibur 4.1 software operated in positive mode. The sample vials were maintained at 10°C in a thermostatic autosampler. Chromatographic separation was achieved on a Halo C18 column (2.1 × 100 mm, 2.7 μm; AMT, USA) with the column temperature set at 45°C. A total of 5 μl of each sample was injected into the column. The mobile phase was composed of water (0.05% formic acid, A) mixed in gradient mode with acetonitrile (0.05% formic acid, B) at a flow rate of 0.3 ml/min. The elution gradient was optimized as follows: 0–1 min 2% B, 1–3 min 2% B to 20% B, 3–4 min 20% B, 4–7 min 20% B to 40% B, 7–9 min 40% B to 70% B, 9–15 min 70% B to 98% B, 15–17 min 98% B. Meanwhile, mass spectrometry detections were set as follows: capillary temperature 300°C; S‐lens RF level 55.0; spray voltage 3.5 kV; sheath gas (N2) flow rate 45 arb; auxiliary gas (N2) flow rate 10 arb; mass spectrometry acquisition range 80–1300 *m/z*; resolution 70,000.

#### 2.4.5 Western blot to detect the protein expression of ERK, JNK, P‐ERK, and P‐JNK

A protein extraction kit was used to extract the total protein from synovial tissue, and a BCA kit was used to detect the protein concentration of the sample. Each group took a sample solution containing the same total protein mass for electrophoresis, transferred the protein to a PVDF membrane, added 5% skimmed milk powder, and blocked for 2 h at room temperature. The dilution multiples of rabbit ERK, JNK, P‐ERK, and P‐JNK primary antibodies were 1:1000, 1:1000, 1:1000, and 1:2000, respectively. The goat anti‐rabbit secondary antibody was diluted at 1:5000, and the color was developed by enhanced chemiluminescence. The gel imaging system was used to take pictures, and the relative expression of each protein group was analyzed by ImageJ software.

### 2.5 Data processing and statistical analysis

Data were collected by using the Xcalibur data system that comes with the instrument. The acquired mass spectrometry data (.raw) were exported into Compound Discover (CD, Thermo Fisher, CA, USA) software for data analysis. CD software converts mass spectrometry data into metabolite information. These metabolic discoveries are achieved through a combination of online open databases and local databases, and MS/MS data of metabolites, which greatly improves the accuracy of metabolite identification. To find the differences between the groups, the data were imported into SIMCA‐P13.0 software (Umetrics, Sweden) for principal component analysis (PCA) and partial least squares discriminant analysis (PLS‐DA), using PLS‐DA for supervised pattern recognition analysis of serum data. Through the CD software, the variables with *p* < .05 and VIP values >1 were screened as difference variables, and the metabolites with mzCloud matching higher than 70 were screened as potential biomarkers. The exact mass of potential biomarkers was searched in databases such as HMDB (http://www.hmdb.ca), METLIN (https://metlin.scripps.edu), and KEGG (http://www.genome.jp/kegg/) for biomarker identification. The identified biomarkers were introduced into metaboanalyst 4.0 (http://www.metaboanalyst.ca) for metabolic pathway analysis, and those whose critical value of metabolic pathway impact was greater than 0.01 were selected as a key potential metabolic pathway to integrate the metabolic pathway and carry out metabolic network analysis to find the core target of *Chaenomeles Fructus* intervention.

Statistical analysis was performed using SPSS 17.0 software, and the experimental data are expressed as the mean ± standard deviation (^−^X ± *SD*). *T* tests were used to compare two groups, and the one‐way analysis of variance (ANOVA) was used to compare differences between multiple groups. *p* < .05 was considered to indicate a significant difference.

## 3 RESULTS

### 3.1 Identification of chemical components in *Chaenomeles Fructus*

**FIGURE 2:**
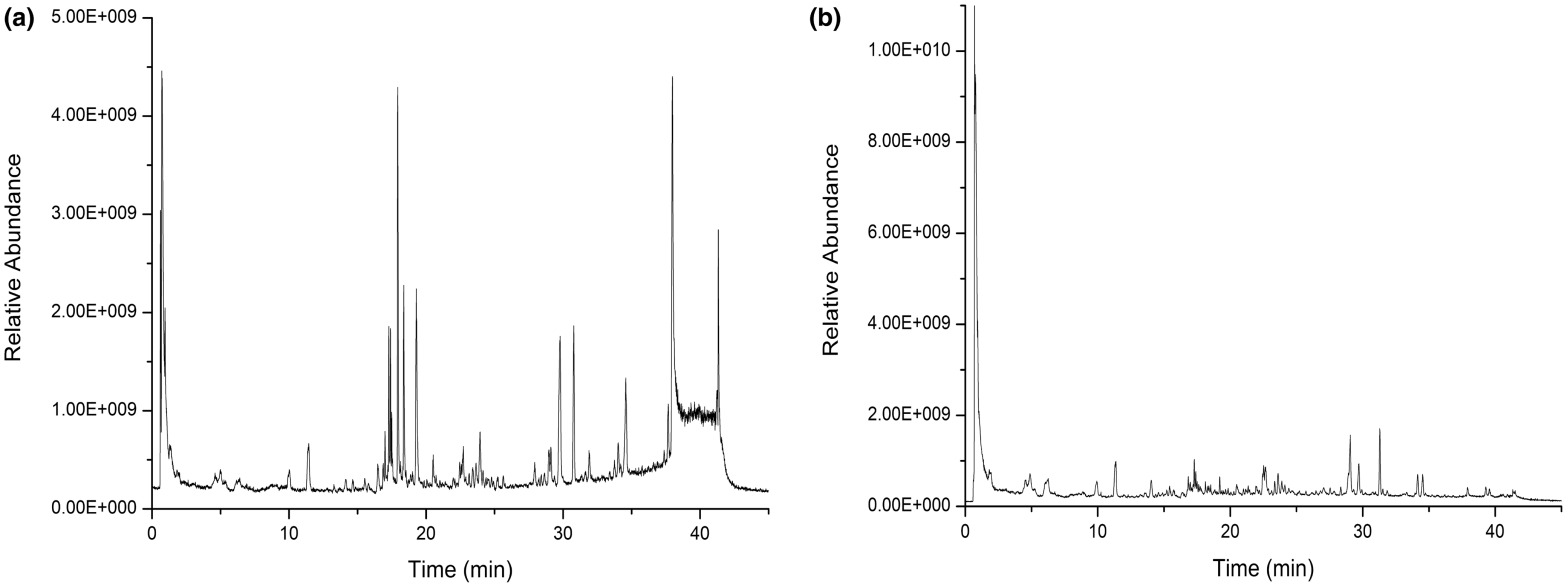
The total ion chromatogram of *Chaenomeles Fructus* extract was collected by mass spectrometry in positive and negative ion modes (a: Positive; B: Negative)

*Chaenomeles Fructus* samples were analyzed under the section “UPLC‐Q‐Exactive Orbitrap MS for Chemical Characterization” chromatographic and mass spectrometry conditions to obtain the UPLC‐Q‐Exactive orbitrap MS total ion current diagram of *Chaenomeles Fructus*, as shown in Figure 2. The results are shown in Table S1.

#### 3.1.1 Amino acids

Compound 1 (Rt 0.66  min) yielded a [M  +  H]^+^ at *m/z* 175.11838, generating 116.07053 [M‐CH_4_N_3_]^+^, 71.06892 [M‐COOH‐CH_4_N_3_]^+^, 70.06566 [M‐CH_2_O_2_‐CH_4_N_3_]^+^, and 60.05628 [M  +  H‐NH_3_‐COOH‐C_4_H_5_]^+^, which was identified as arginine (Qu et al., 2008). Compound 4 (Rt 0.74  min) was identified as valine, showing main fragmentations at *m/z* 72.08121 [M‐COOH]^+^ and 58.01757 [M  +  H‐COOH‐NH_3_]^+^ (Qu et al., 2008). Compound 5 showed [M  +  H]^+^ at *m/z* 182.08064 (C_9_H_11_NO_3_) with key fragmentations at *m/z* 165 and 119. It was identified as tyrosine (Zhang, Dong, et al., 2017). Compound 6 was leucine, which showed characteristic ions at *m/z* 86.09675 [M‐COOH]^+^, 69.03406 [M‐COOH‐NH_3_]^+^, and 57.03419 [M  +  H‐NH_3_‐CH]^+^ (Cao et al., 2004; Qu et al., 2008).

#### 3.1.2 Organic acids

In negative‐ion mode, the retention time of organic acids is 0.71  ~  33.34  min. Compound 8 (Rt 0.97  min) yielded a [M‐H]^−^ at *m/z* 191.05516, producing 173.00809 [M‐H‐H_2_O]^−^, 85.02785 [M‐H‐C_3_H_5_O_3_]^−^, which was identified as quinic acid (Wang et al., 2010). Compound 9 (Rt 1.03  min) yielded a [M‐H]^−^ at *m/z* 117.01778, generating 99.00713 [M‐H‐H_2_O]^−^, 73.02792 [M‐H‐CO_2_]^−^, 55.01737 [M‐H‐H_2_O‐CO_2_]^−^ and was confirmed with a standard and thus identified as succinic acid (Zhang, Dong, et al., 2017). Compound 11 was identified as gallic acid, showing [M‐H]^−^ at 169.01299 (C_7_H_6_O_5_). The key fragment of *m/z* 125.02288 indicates that there is a carboxyl group in the A ring. Compound 13 (Rt 1.96  min) yielded a [M‐H]^−^ at *m/z* 153.01804, producing 109.02789 [M‐H‐CO_2_]^−^, 91.01732 [M‐H‐CO_2_‐H_2_O]^−^, 81.49275 [M‐H‐C_2_O_3_]^−^, which was identified as protocatechuic acid (Yu et al., 2016). Compound 14 was detected at 1.99  min with a calculated formula of C_7_H_10_O_5_. Its characteristic fragments included ions at *m/z* 155.03384 [M‐H‐H_2_O]^−^, 137.02292 [M‐H‐H_2_O‐H_2_O]^−^, 111.04358 [M‐H‐C_2_H_2_O_3_]^−^, and 93.03295 [M‐H‐CH_4_O_4_]^−^, which were identical to those of shikimic acid. Compound 16 ([M‐H]^−^, *m/z* 137.02299) underwent successive loss of H_2_O (18  Da) and CO (28  Da) and was identified as 4‐hydroxybenzoic acid (Gao et al., 1999). 2‐isopropylmalic acid (17) eluting at 3.64  min showed an [M‐H]^−^ ion at *m/z* 175.06000 (C_7_H_12_O_5_), which was identical to the reference standard. Compound 20 was identified as chlorogenic acid, showing main fragmentations at *m/z* 191.05508 [M‐H‐C_9_H_7_O_3_]^−^, 173.04468 [M‐H‐C_9_H_9_O_4_]^−^, and 161.02345 [M‐H‐C_9_H_8_O_4_]^−^ (Ju et al., 2007). Compound 21 was caffeic acid, which showed characteristic ions at m/z 135.04362 [M‐H‐CO_2_]^−^ and 107.04925 [M‐H‐C_2_O_3_]^−^ (Yu et al., 2016). Compound 23 was identified as vanillic acid, producing the main fragmentations at *m/z* 153.01799[M‐H‐CH_2_]^−^, 123.04520[M‐H‐COO]^−^, and 109.02951[M‐H‐CH_2−_COOH]^−^. Compound 24 showed [M‐H]^−^ at *m/z* 165.05443 (C_9_H_10_O_3_) with key fragment ions at *m/z* 148 and 71. It was identified as L‐3‐phenyl lactic acid. Compound 25 eluted at 11.07  min displayed [M‐H]^−^ at m/z 367.10294, with main fragmentations at m/z 191.05502, 179.03419, 135.03912, and 93.03273. The fragment at m/z 191.05502 was due to the loss of C_10_H_8_O_3_, m/z 179.03419 was due to the loss of C_8_H_12_O_5_, and m/z 135.03912 and 93.032 73 corresponded to C_9_H_12_O_7_ and C_11_H_14_O_8_, respectively. It was identified as methyl chlorogenate (Liu et al., 2016). Compound 40 was identified as linoleic acid, showing main fragmentations at m/z 263.23584 [M  +  H‐H_2_O]^+^, 221.22533 [M‐COOH‐CH_2_]^+^, 179.14233 [M‐COOH‐C_4_H_8_]^+^_,_ and 165.12689 [M‐COOH‐C_5_H_10_]^+^ (Hou et al., 2015). Compound 46 (Rt 33.34  min) yielded a [M‐H]^−^ at m/z 237.22162 [M‐H‐H_2_O]^−^, 211.24319 [M‐H‐CO_2_]^−^, and 197.15478 [M‐H‐C_4_H_10_]^−^, which was identified as palmitic acid. Other organic acids included 2 (tartaric acid), 3 (malic acid), 7 (citric acid), 10 (D‐α‐hydroxyglutaric acid), 12 (kojic acid), 15 (fumaric acid), 22 (benzoic acid), and 26 (cinnamic acid). The retention time, MS, and MS2 data of 2, 3, 7, 10, 12, 15, 23, and 28 were identical to the reference standards.

#### 3.1.3 Flavonoids

Compound 34 was identified as Luteolin, showing main fragmentations at m/z 243 [M‐H‐C_2_H_2_O]^−^, 241 [M‐H‐CO_2_]^−^, 199 [M‐H‐C_2_H_2_O‐CO_2_]^−^, 217 [M‐H‐C_3_O_2_]^−^, and 175 [M‐H‐C_2_H_2_O‐C_3_O_2_]^−^. Compound 27 eluted at 14.26  min and displayed [M‐H]^−^ at m/z 577.15628, with main fragmentations at m/z 413.08788 and 297.04053. The fragment at m/z 413.08788 was due to the loss of C_6_H_12_O_5_, and the fragment at m/z 297.04053 was due to the loss of C_6_H_12_O_5_ and C_5_H_8_O_3._ It was identified as vitexin‐2‐O‐rhamnoside. Compounds 28, 29, 31, and 35 displayed molecular ions at m/z 609.14611, 463.08820, 433.07763, and 447.09328, respectively, which produced a common fragment at m/z 301. The losses of 308, 162, 132, and 146  Da corresponded to residues of rutinose, galactose, arabinose, and rhamnose, respectively. Compounds 28, 29, 31, and 35 were thus identified as rutin (kaempferol‐3‐O‐rutinoside), hyperoside (quercetin‐3‐O‐galactoside), quercetin‐3‐O‐arabinofuranoside, and quercitrin (kaempferol‐3‐O‐rhamnopyranoside), respectively. Compound 37 ([M‐H]^−^, m/z 271.06131) underwent successive losses of C_9_H_8_O_3_ (164  Da), C_8_H_8_O (120  Da), and C_5_H_2_O_2_ (94  Da) and was identified as naringenin (Sun et al., 2020). Compound 33 showed an m/z [M‐H]^−^ at 433.11414 (C_21_H_22_O_10_). The key fragment of m/z 271.06113 indicates that there is a glucose group in the structure. Compound 33, having similar fragmentations to 37, was identified as naringenin‐7‐O‐glucoside. Compound 38 (Rt 18.16  min) yielded a [M‐H]^−^ at m/z 257.04504 [M‐H‐CO]^−^, 229.05028 [M‐H‐2CO]^−^, 185.05972 [M‐H‐2CO‐CO_2_]^−^, and 151.00232 [M‐H‐C_8_H_6_O_2_]^−^, 133.02953 [M‐H‐C_7_H_4_O_4_]^−^, which was identified as kaempferol (Bai et al., 2018). Other flavonoids included 18 (catechin), 30 (vitexin), 32 (vicenin), and 36 (quercetin). The retention time, MS, and MS2 data of 18, 32, 34, and 37 were identical to the reference standards.

#### 3.1.4 Triterpenoids

A total of seven triterpenoids (39, 41, 42, 43, 44, 45, 47, and 48) were found in *Chaenomeles Fructus*. Compound 39 was detected at 21.93  min with a calculated formula of C_30_H_48_O_4_. Its characteristic fragments included ions at m/z 453.34192 [M‐H‐H_2_O]^−^ and 407.33151 [M‐H‐H_2_O‐CO_2_]^−^, which were identical to those of maslinic acid (Wang et al., 2017). Compound 41 was identified as 3‐O‐acetyl pomolic acid, showing main fragmentations at m/z 495.35403 [M‐H‐H_2_O]^−^ and 453.14035 [M‐H‐H_2_O‐C_2_H_2_O]^−^. Compound 42 showed [M  +  H]^+^ at m/z 457.36600 (C_30_H_48_O_3_) with key fragment ions at m/z 439, 411, and 397. It was identified as betulinic acid. Compound 43 (Rt 29.46  min) yielded a [M  +  H]^+^ at m/z 439.35739 [M  +  H‐H_2_O]^+^, 411.36209 [M  +  H‐H_2_O‐CO]^+^, and 249.18672 [M  +  H‐C_14_H_24_O]^+^, which was identified as oleanolic acid (Wang et al., 2017). Compound 44 was betulin, which showed characteristic ions at m/z 425.37711 [M  +  H‐H_2_O]^+^, 407.29532 [M  +  H‐2H_2_O]^+^, and 221.19011 [M  +  H‐C_15_H_26_O]^+^ (Huo et al., 2016). Compound 45 (Rt 31.98  min) yielded a [M  +  H]^+^ at m/z 425.37622 [M  +  H‐H_2_O]^+^ and 395.36612[M  +  H‐H_2_O‐CH_2_O]^+^, which was identified as erythrodiol. Compound 47 ([M  +  H]^+^, m/z 427.39383; C_30_H_50_0) had key fragments at m/z 409, 260, and 191. They were identified as β‐amyrin. Ursolic acid (48) eluting at 34.55  min showed a [M‐H]^−^ at m/z 455.35269 (C_30_H_48_O_3_), which was identical to the reference standard.

#### 3.1.5 Others

Escinolide (19) eluting at 4.77 min showed a [M‐H]^−^ at m/z 177.01933 (C_9_H_6_O_4_), which was identical to the reference standard.

### 3.2 Network construction

**FIGURE 3:**
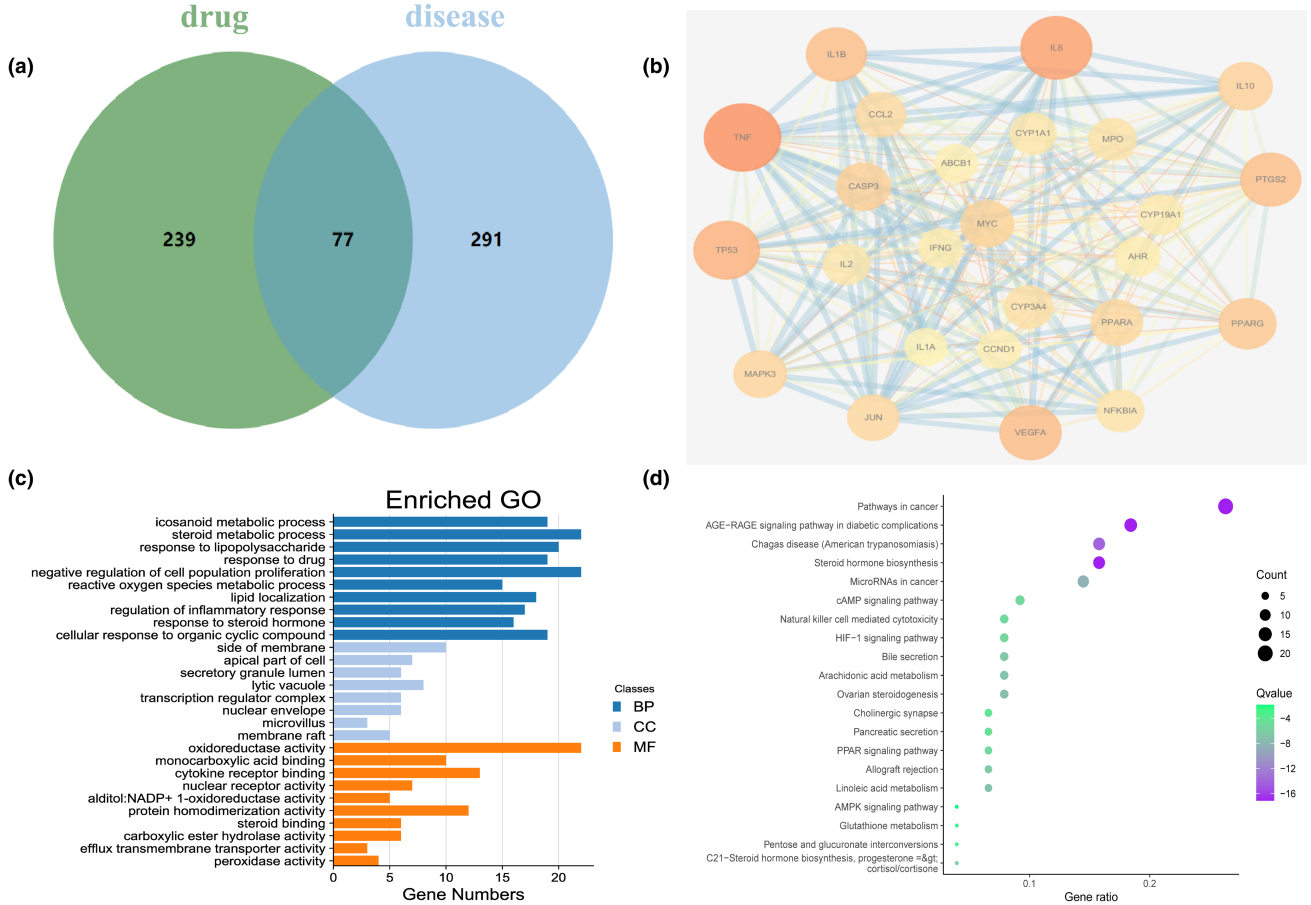
The intersection of compounds and gene targets. The intersection showed 77 genes between UPLC‐Q‐Exactive orbitrap MS analysis and RA‐related genes, green circle represented the target genes of *Chaenomeles Fructus*, and the blue circle represented RA‐related genes. (b) The PPI network of the target of *Chaenomeles Fructus* on RA (the larger the area of the target, the darker the corresponding color, indicating that the deeper the connection between this target and other targets; the wider the width of the line between targets, the darker the corresponding color, indicating that the higher the degree of connection between the two targets; degree score > 21). (c) Analysis of GO enrichment of *Chaenomeles Fructus* on RA. The GO enrichment analysis contained the top 10 biological processes (dark blue), molecular function (orange), and 8 cellular components (light blue). (d) KEGG analysis of the *Chaenomeles Fructus* on RA

Using the 48 identified compounds to find their targets in TCMSP and Swiss TargetPrediction, a total of 316 targets were obtained and named according to their gene symbols, and a total of 7 compounds were screened (Table 1). We used “rheumatoid arthritis” as the search term to construct related targets of RA in the TTD and OMIM databases and then merged the gene targets retrieved from the two databases, removed duplicate values, and took their union. A total of 368 related genes were identified. The Venny 2.1.0 tool (https://bioinfogp.cnb.csic.es/tools/venny/) was used to map and compare the target genes of *Chaenomeles Fructus* with the RA genes to obtain the intersection of 77 target genes (Figure 3a). Then, they were imported into the STRING database and Cytoscape 3.7.1 to construct a PPI network to obtain protein interaction relationships (Figure 3b). The result of the screening degree score greater than 21 shows that there are 25 nodes and 238 edges in the network, and TNF, IL6, IL1B, VEGFA, MAPK3, etc., are its main targets. GO function annotation includes three aspects: cell component, molecular function, and biological process. Cell components mainly involved the side of the membrane, apical part of the cell, and secretory granule lumen; molecular function was mainly coupled with oxidoreductase activity, monocarboxylic acid binding, and cytokine receptor binding; and the biological process mainly involved the icosanoid metabolic process, steroid metabolic process, response to lipopolysaccharide, and regulation of inflammatory response. (Figure 3c). The top 20 significantly enriched pathways are shown in Figure 3d. Among these potential pathways, the AGE‐RAGE signaling pathway, cAMP signaling pathway, HIF‐1 signaling pathway, and arachidonic acid metabolism were also included, which were categorized as related to inflammation. After integrating drug target prediction, pathway and function enrichment, and network analyses, we identified TNF, IL6, IL1B, VEGFA, and MAPK3 as relatively highly relevant targets in inflammation. Additionally, they are considered the key targets of *Chaenomeles Fructus* in the treatment of RA. Thus, we speculated that *Chaenomeles Fructus* may interfere with inflammation by inhibiting the release of inflammatory factors and inflammatory signaling pathways to treat RA.

**TABLE 1:** Compounds of *Chaenomeles Fructus* screened by TCMSP

No.	Compounds	Molecular formula	Molecular weight	Molecule ID	OB(%)	DL
1	Catechin	C_15_H_14_O_6_	289.07	MOL000492	54.83	0.24
2	Quercetin	C_15_H_10_O_7_	301.04	MOL000098	46.43	0.28
3	Escinolide	C_9_H_6_O_4_	178.14	MOL004456	30.43	0.36
4	Naringenin	C_15_H_12_O_5_	271.06	MOL004328	59.29	0.21
5	Luteolin	C_15_H_10_O_6_	286.25	MOL000006	36.16	0.25
6	Kaempferol	C_15_H_10_O_6_	286.25	MOL000422	41.88	0.24
7	Betulinic acid	C30H48O3	456.70	MOL000211	55.38	0.78

### 3.3 Experimental validation

#### 3.3.1 CFA‐injected rats

**FIGURE 4:**
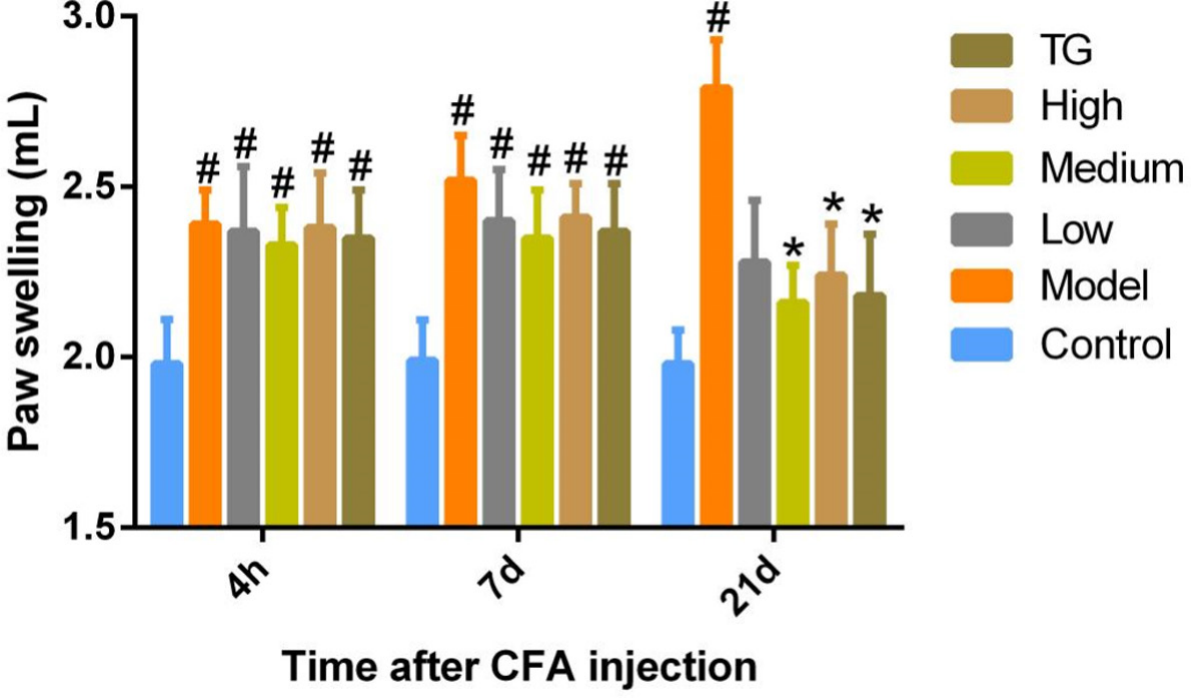
Effects of *Chaenomeles Fructus* alcohol extract on the degree of foot swelling in RA rats. Data are presented as mean ± *SD*, *n* = 10. ^#^ *p* < .05, vs. control group, *p < .05, vs. model group

During the experiment, it was observed that the rats were easy to move, and the sizes of the left and right feet were similar before modeling. After modeling for 4  h, the rats developed swelling of the right toe and limited movement, especially the arthritic symptoms of the right foot, which indicated that the model was successful. There was no significant change in the volume of the toes of the rats in the control group, and the rats were active. The swelling of the right foot of the rats in each dose group of *Chaenomeles Fructus* and TG group was significantly relieved after administration (Figure 4). Compared with the model group, the swelling degree of the rat toes in the high‐ and medium‐dose groups was significantly reduced after 21  days.

#### 3.3.2 Multivariate statistical analysis of metabolomics data

**FIGURE 5:**
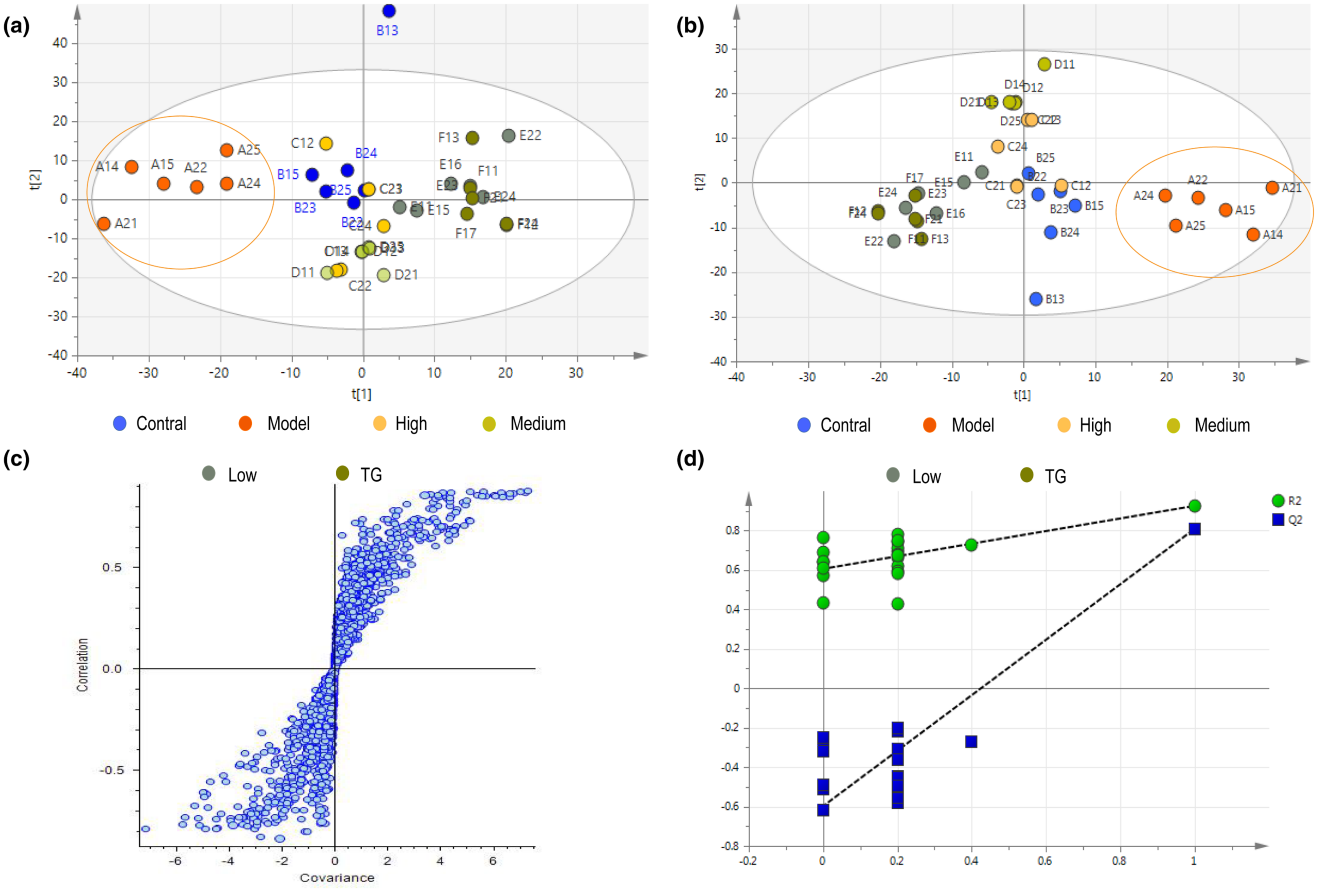
PCA scores plot [R2X (cumulative), 0.579], the red circle represents the model group; (b) PLS‐DA score plots [R2Y (cumulative), 0.879; Q2 (cumulative), 0.588], the red circle represents the model group; (c) S‐plot showing metabolites with significant differences between groups; (d) 100 permutations of the PLS‐DA model [R2 = 0.607, Q2 = ‐0.598]; *n* = 11 per group

All quality control (QC) samples were pooled together and concentrated at a 95% confidence interval, which indicates that the instrument is working properly and that the data quality is reliable. The metabolites were discovered with significant differences between the groups shown by PCA (Figure 5a), PLS‐DA (Figure 5b), and S‐plot (Figure 5C). The results of the PLS‐DA model showed an obvious separation trend among all groups; the samples of each group were separated from each other and clustered together individually. The model group and the control group were separated clearly, which indicated that the animal model was successful, and the occurrence of disease could cause significant changes in endogenous metabolites. 100 permutation tests (Figure 5d) were used to verify the accuracy of the model.

#### 3.3.3 Potential biomarkers

Through the constructed PLS‐DA model, the metabolic ions that deeply influenced the clustering trend were selected. Metabolites of VIP value >1 were selected. Verification was performed on variables selected by testing two independent‐samples T. Differential metabolite data were evaluated as significant at *p* < .05 based on the Compound Discoverer 2.0 software database, and MZ Cloud, ChemSpider, Variable discrepancy identification was achieved by adapting high‐resolution MS spectra and MS/MS spectra combined with secondary fragmentations. The metabolite discrepancy data achieved in the verification were analyzed through MetaboAnalyst 3.0 for enrichment analysis. Referring to relevant literature reports, related metabolic pathways involved in discrepant metabolites were analyzed by using the KEGG online database. Enrichment analysis and analysis of the relevant metabolic pathways involved in differential metabolites using the KEGG online database and related. The results are shown in Table S2.

#### 3.3.4 Metabolic pathway analysis

**FIGURE 6:**
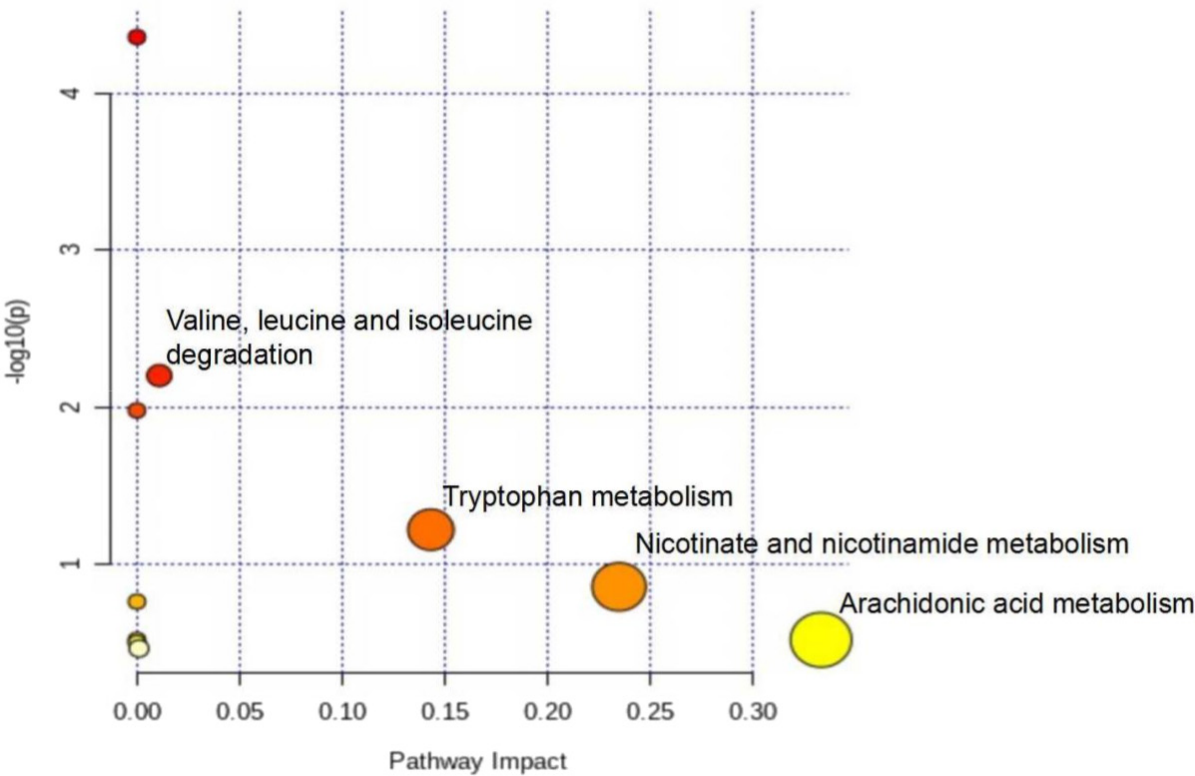
Principal metabolic pathways of *Chaenomeles Fructus* for the treatment of RA

The differentially produced metabolites were entered into Metaboanalyst (http://www.metaboanalyst.ca) for enrichment analysis of the metabolic pathways. Metabolic pathway analysis found that *Chaenomeles Fructus* mainly interferes with arachidonic acid metabolism, nicotinate and nicotinamide metabolism, tryptophan metabolism, and branched‐chain amino acid catabolism (valine, leucine, and isoleucine degradation) (Figure 6).

#### 3.3.5 Effect of Chaenomeles Fructus alcohol extract on the protein expression of ERK, JNK, P‐ERK, and P‐JNK in the synovium of RA model rats

**FIGURE 7:**
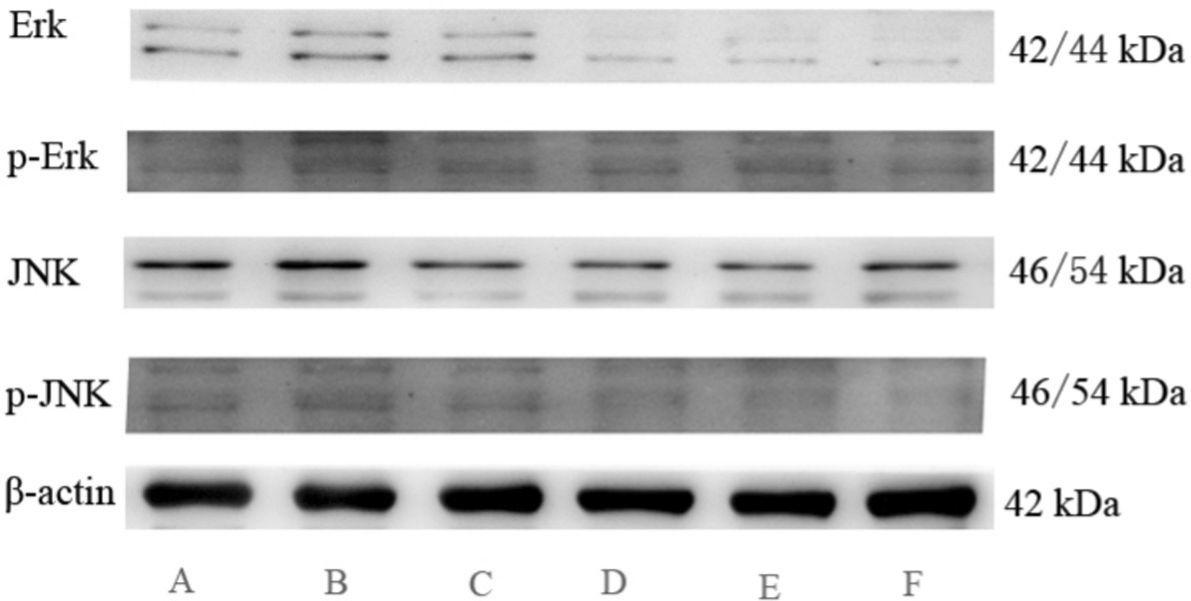
Effects of alcohol extract separated from *Chaenomeles Fructus* on levels of protein expression of ERK, JNK, P‐ERK, and P‐JNK in RA model rats. (A) Control, (B) model, (C) low, (D) medium, (E) high, (F) TG

The protein expression results are shown in Figure 7 and Table 2. The expression of ERK, JNK, P‐ERK, and P‐JNK proteins related to inflammation increased, but their expression levels showed significant differences compared with the control group (*p*  <  0.01). After the intervention, the expression levels of ERK, JNK, and P‐JNK decreased, which were significantly different from the model group (*p*  <  0.01).

**TABLE 2:** Effects of alcohol extract separated from *Chaenomelis Fructus* on levels of ERK, JNK, P‐ERK, and P‐JNK protein expression in RA model rats

Groups	ERK	P‐ERK	JNK	P‐JNK
Control	0.29 ± 0.02	0.13 ± 0.01	0.22 ± 0.05	0.12 ± 0.01
Model	0.78 ± 0.04^##^	0.28 ± 0.01^##^	0.63 ± 0.09^##^	0.34 ± 0.07^##^
Low	0.45 ± 0.05	0.26 ± 0.02	0.60 ± 0.12	0.29 ± 0.05
Medium	0.34 ± 0.09^##△△^	0.34 ± 0.09^##△△^	0.41 ± 0.04^△△^	0.22 ± 0.05^##△△^
High	0.31 ± 0.01^##△△^	0.31 ± 0.01^##△△^	0.26 ± 0.02^△△^	0.16 ± 0.02^##△△^
TG	0.31 ± 0.02^##△△^	0.31 ± 0.02^##△△^	0.25 ± 0.06^△△^	0.13 ± 0.03^##△△^

*Note*: (^##^ *p* < .01, vs. group Control; ^△△^ *p* < .01, vs. group Model).

## 4 DISCUSSION

In recent years, the application of liquid‐mass spectrometry technology in the identification of multicomponent medicinal plants and their compound ingredients has become increasingly extensive (Li et al., 2018; Liu et al., 2017; Yin et al., 2020), but research on the medicinal plant *Chaenomeles Fructus* mainly uses gas‐mass spectrometry to analyze the volatile oil of *Chaenomeles Fructus* (Wang, Zhang, et al., 2020; Zhang, Jiang, et al., 2017), while the liquid‐mass spectrometry analysis of the nonvolatile oil components of *Chaenomeles Fructus* is less common. This study used UPLC‐Q‐Exactive orbitrap MS technology to identify the chemical components of 48 *Chaenomeles Fructus*, including four types: amino acids, organic acids, flavonoids, and triterpenes.

The common animal models of RA mainly include adjuvant‐induced arthritis (AA), collagen II‐induced arthritis (CIA), streptococcal cell wall‐induced arthritis (SCW), and pristane‐induced arthritis (PIA). The AA model is more in line with the economic principle, the construction method of the model is relatively simple, and the success rate of modeling is high (Li, Li, & Li, 2021; Li, Zhang, et al., 2021). The CIA modeling process is cumbersome, and the preparation of the immune mixture requires the entire operation on ice (Li, Li, & Li, 2021). The economic cost of SCW model inducers is relatively high, and the intra‐articular injection technique is difficult. The peptidoglycan‐polysaccharide (PG‐PS) structure of the different Streptococcus cell walls and the degree of development of the SCW are also different, which leads to the frequency of use of the SCW model in the study being low (Cromartie et al., 1977; Marijnissen et al., 2014). The course of PIA is less invasive, but the construction of the model is time‐consuming (Hutamekalin et al., 2009; Song et al., 2015). In this study, the CFA‐induced AA model was used to investigate the therapeutic effect of *Chaenomeles Fructus* alcohol extract on RA. An immune agent, CFA, is used to stimulate the animal model to form immune hyperactive joint inflammation. The pathological changes were very similar to those of RA in clinical practice (Chen et al., 2019; Honmore et al., 2019; Tang et al., 2021; Zhu, 2016). Paw swelling is a typical symptom of arthritic rats and is measured to determine the anti‐inflammatory activity of various drugs. Our results showed that the high‐, medium‐, and low‐dose *Chaenomeles Fructus* alcohol extracts reduced toe swelling in CFA‐induced AA model rats to varying degrees. It indicates that the alcohol extract of *Chaenomeles Fructus* has a good anti‐inflammatory effect and has a definite therapeutic effect on the treatment of RA.

The results of the PPI network revealed that the core targets of *Chaenomeles Fructus* in the treatment of RA are TNF, IL6, IL1B, VEGFA, MAPK3, etc. TNF, IL‐6, and IL‐1B, as proinflammatory cytokines, can stimulate the inflammation and degradation of bone and cartilage and gradually lead to arthritis damage and deformity in RA patients and finally disability (Luo et al., 2022). VEGF can significantly improve the permeability of blood vessels in patients, can promote the formation and development of inflammation, and is of great significance to the formation of chronic inflammation. The serum VEGF level is an important indicator for judging the condition and prognosis of RA (Yu et al., 2018). MAPK3, known as ERK1, is involved in many pathogenic processes of RA, including its role in promoting the expression of inflammatory cytokines (Bauer et al., 2000). Combined with GO function and KEGG pathway enrichment analysis, the intersection target mainly involves the AGE‐RAGE signaling pathway, cAMP signaling pathway, HIF‐1 signaling pathway, arachidonic acid metabolism, and other inflammatory response regulation pathways. Studies have shown that AGE‐RAGE can stimulate the production of proinflammatory factors. It can also act as an inflammatory factor to activate innate immune cells and further lead to the development of arthritis (Millerand et al., 2019). As an important second messenger in cells, cAMP's main role is to bind to PKA regulatory subunits, thereby activating PKA. The increase in the content of cAMP and the activation of PKA eventually cause the activation of NF‐κB and the increase in the expression of the proinflammatory cytokine IL‐6, which are thought to be associated with RA disease progression (Ilchovska & Barrow, 2021; Narasimamurthy et al., 2012; Wu et al., 2013). HIF‐1α is a very important transcriptional regulator in the mammalian body under hypoxic conditions. Studies have shown that NF‐κB can promote the secretion of inflammatory factors by macrophages in a HIF‐1α‐dependent and HIF‐1α‐independent manner, thereby inducing the occurrence of RA (Knowles et al., 2006). The arachidonic acid (AA) metabolic pathway is an important metabolic pathway in the inflammatory response and is highly activated in the inflammatory response. When cells are stimulated, the cell membrane phospholipase A2 (PLA2) releases AA (Yu et al., 2022). The metabolite of arachidonic acid, prostaglandin E2 (PGE2), is a major mediator of inflammation in diseases such as rheumatoid arthritis and osteoarthritis (Park et al., 2006). It can be seen that *Chaenomeles Fructus* mainly alleviates RA from an anti‐inflammatory perspective.

There are many signaling pathways involved in inflammation, and the inhibition of the MAPK signaling pathway is an important means to effectively control the occurrence of inflammation. MAPK mainly includes three major pathways: JNK, ERK1/2, and p38. JNK can be activated by inflammatory factors such as TNFα and IL‐1β. ERK1/2 can promote the expression of inflammatory cytokines, and p38 can promote the expression and secretion of inflammatory factors in cells (Bauer et al., 2000; Luo et al., 2022). In this study, the alcohol extract of *Chaenomeles Fructus* reduced the protein expression of JNK and ERK1/2 protein in the synovium of the knee joint of RA rats, suggesting that the alcohol extract of *Chaenomeles Fructus* can inhibit the MAPK signaling pathway, thus reducing the release of inflammatory factors and inhibiting the abnormal proliferation of the synovial membrane to inhibit joint bone erosion in RA.

Based on the metabolomics research method, this experiment constructed the overall metabolic network of *Chaenomeles Fructus* intervention for RA by integrating the body's metabolic pathways. *Chaenomeles Fructus* interferes with the metabolism of inflammatory factors by downregulating arachidonic acid metabolism and branched‐chain amino acid catabolism, thereby inhibiting the production of inflammatory factors and reducing the inflammatory response. Arachidonic acid is a long‐chain polyunsaturated fatty acid and an important mediator that regulates inflammation (Higgins & Lees, 1984; Sala et al., 2018). It is involved in the metabolism of arachidonic acid and the synthesis and release of inflammatory factors such as tumor factors and interleukins (Lewis et al., 1990). BCAAs include leucine, isoleucine, and valine. Supplementation with BCAAs could activate mTORC1 and upregulate the NF‐κB signaling pathway, increasing the release of proinflammatory cytokines in human peripheral blood mononuclear cells and endothelial cells (Ye et al., 2020; Zhenyukh et al., 2017, 2018). In the model group of this study, the content of arachidonic acid and BCAAs in the serum was upregulated, suggesting that there is an inflammatory reaction in the serum of RA rats. After administration of *Chaenomeles Fructus* alcohol extract, the contents of arachidonic acid and BCAAs were decreased, indicating that *Chaenomeles Fructus* could interfere with the metabolism of arachidonic acid and BCAAs in RA rats, thus affecting the inflammatory reaction in which arachidonic acid and BCAAs were involved and producing anti‐inflammatory effects.

**FIGURE 8:**
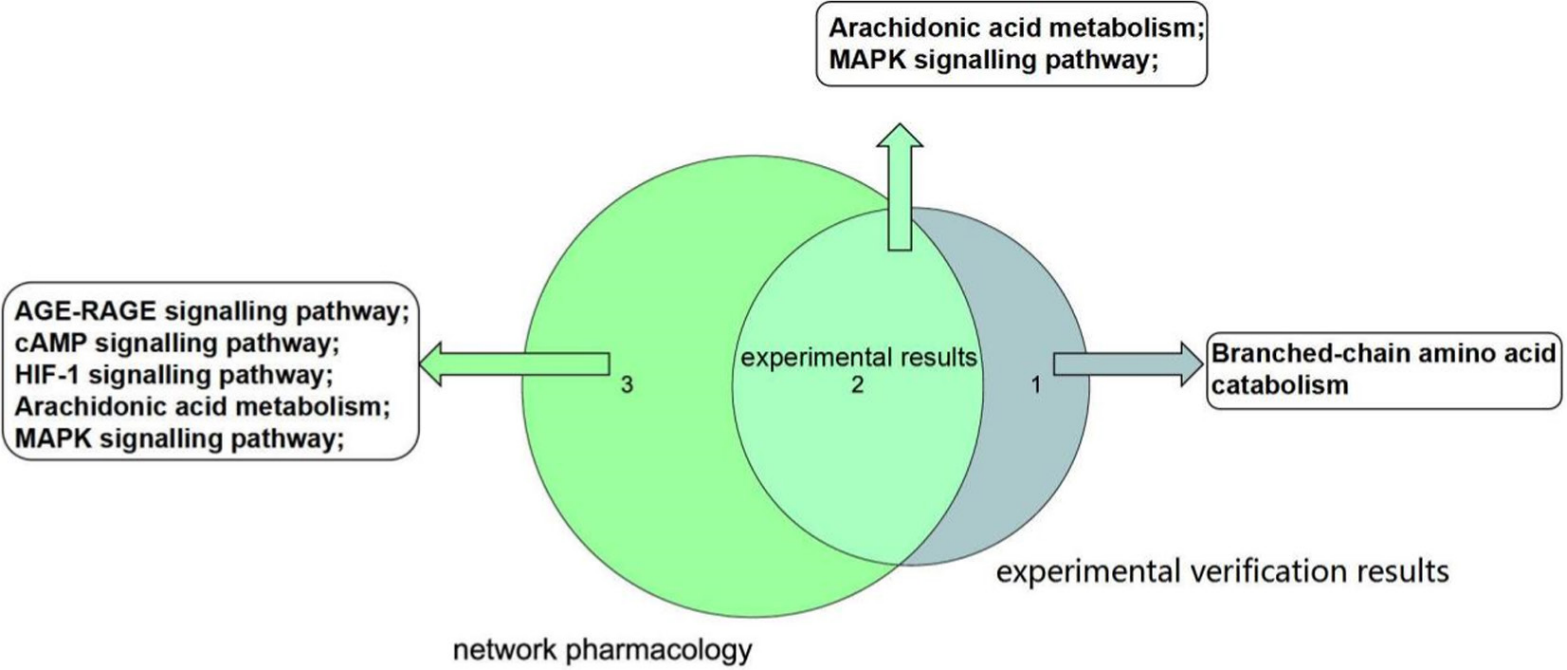
Venn analysis of network pharmacology and experimental verification results

The integrated network pharmacology results showed that *Chaenomeles Fructus* mainly relieves RA by interfering with the MAPK signaling pathway, AGE‐RAGE signaling pathway, cAMP signaling pathway, HIF‐1 signaling pathway, arachidonic acid metabolism, and other inflammatory pathways. Inflammation plays an important role in the pathological process of RA. Then, we used CFA‐induced RA rats to carry out the verification test. Combining MAPK signaling pathway proteins and serum nontargeted metabolomics results, it was concluded that *Chaenomeles Fructus* mainly interferes with the metabolism of inflammatory factors through arachidonic acid metabolism, the MAPK signaling pathway, and branched‐chain amino acid catabolism to inhibit the production of inflammatory factors and reduce the inflammatory response. In summary, the results of the network pharmacology prediction and the verification experimental results were analyzed by Venn analysis, and the experimental results were concluded; that is, *Chaenomeles Fructus* mainly interferes with the inflammation of RA by inhibiting arachidonic acid metabolism and the MAPK signaling pathway (Figure 8).

## 5 CONCLUSION

Taking all the above results together, we identified 48 chemical components from *Chaenomeles Fructus* based on UPLC‐Q‐Exactive orbitrap MS, which mainly includes four types: amino acids, organic acids, flavonoids, and triterpenes. Moreover, we predicted the mechanism of action of *Chaenomeles Fructus* in the treatment of RA with network pharmacology and verified it through CFA‐induced RA in rats. The results of the network pharmacology prediction and the verification experimental results were analyzed by Venn analysis, and the experimental results concluded that *Chaenomeles Fructus* mainly interferes with the inflammation of RA by inhibiting arachidonic acid metabolism and the MAPK signaling pathway.

## CONFLICT OF INTEREST

The authors declare that there are no conflicts of interest.

## ETHICS APPROVAL AND CONSENT TO PARTICIPATE

All animal welfare and experimental procedures were performed in accordance with the National Institutes of Health Guide for the Care and Use of Laboratory Animals, and the protocols used were approved by the Animal Ethics Committee of Shandong University of Traditional Chinese Medicine Laboratory Animal Center, Jinan, China.

## Supporting information


Tables S1 and S2

## Data Availability

The data that support the findings of this study are available from the corresponding author upon reasonable request.
