# Supplementary material for: Deciphering the pharmacological mechanisms of Chaenomeles Fructus against rheumatoid arthritis by integrating network pharmacology and experimental validation
Source: Food Sci Nutr. 2022 Jul 7;10(10):3380–94. doi: 10.1002/fsn3.2938 (PMC9548373; doi:10.1002/fsn3.2938)
Supplement: Supplementary file 1 — Tables S1 and S2 [file FSN3-10-3380-s001.doc]

**Supplement Table 1. Identified compounds from Chaenomelis Fructus by UPLC-Q-Exactive orbitrap MS**

| No. | Retention time(min) | Identified compounds | Molecular formula | [M-H] | | | MS^2^ data (m/z) | [M+H] | |  | MS^2^ data (m/z) |
| --- | --- | --- | --- | --- | --- | --- | --- | --- | --- | --- | --- |
|  |  |  |  | Detection value(m/z) | Theoretical value(m/z) | Error (ppm) |  | Detection value(m/z) | Theoretical value(m/z) | Error (ppm) |  |
| 1 | 0.66 | Arginine | C_6_H_14_N_4_O_2_ |  |  |  |  | 175.11838 | 175.11895 | -0.00057 | 116.07053、71.06892、70.06566、60.05628 |
| 2 | 0.71 | Tartaric acid | C_4_H_6_O_6_ | 149.00774 | 149.00916 | -0.00142 | 130.99670、113.02288、84.99312 |  |  |  |  |
| 3 | 0.73 | Malic acid | C_4_H_6_O_5_ | 133.01280 | 133.01425 | -0.00145 | 130.99861、115.00214、86.00094 |  |  |  |  |
| 4 | 0.74 | Valine | C_5_H_11_NO_2_ |  |  |  |  | 118.08592 | 118.08626 | -0.00034 | 72.08121、58.01757 |
| 5 | 0.77 | Tyrosine | C_9_H_11_NO_3_ |  |  |  |  | 182.08064 | 182.08117 | -0.00053 | 165.05408、119.04877 |
| 6 | 0.90 | Leucine | C_6_H_13_NO_2_ |  |  |  |  | 132.10120 | 132.10191 | -0.00071 | 86.09675、69.03406、57.03419 |
| 7 | 0.93 | Citric Acid | C_6_H_8_O_7_ | 191.01868 | 191.01973 | -0.00105 | 173.00790、129.01781、 |  |  |  |  |
| 8 | 0.97 | Quinic acid | C_7_H_12_O_6_ | 191.05516 | 191.05611 | -0.00095 | 173.00809、85.02785 |  |  |  |  |
| 9 | 1.03 | Succinic acid | C_4_H_6_O_4_ | 117.01778 | 117.01933 | -0.00155 | 99.00713、73.027 92  55.017 37 |  |  |  |  |
| 10 | 1.09 | D-α-Hydroxyglutaric acid | C_5_H_8_O_5_ | 147.02844 | 147.02990 | -0.00146 | 129.01782、111.00716 |  |  |  |  |
| 11 | 1.15 | Gallic acid | C_7_H_6_O_5_ | 169.01299 | 169.01425 | -0.00126 | 151.05978、125.02288 |  |  |  |  |
| 12 | 1.91 | Kojic acid | C_6_H_6_O_4_ | 141.01773 | 141.01933 | -0.0016 | 113.02288、93.03296 |  |  |  |  |
| 13 | 1.96 | Protocatechuic acid | C_7_H_6_O_4_ | 153.01804 | 153.01933 | -0.00129 | 109.02789、91.01732、81.49275 |  |  |  |  |
| 14 | 1.99 | Shikimic acid | C_7_H_10_O_5_ | 173.04446 | 173.04555 | -0.00109 | 155.03384、137.02292、111.04358、93.03295 |  |  |  |  |
| 15 | 2.92 | Fumaric acid | C_4_H_4_O_4_ | 115.00211 | 115.00368 | -0.00157 | 96.96794、87.00723 |  |  |  |  |
| 16 | 3.01 | 1. Hydroxybenzoic acid | C_7_H_6_O_3_ | 137.02299 | 137.02442 | -0.00143 | 119.01260、108.02033 |  |  |  |  |
| 17 | 3.64 | 1. isopropylmalic acid | C_7_H_12_ O_5_ | 175.06000 | 175.06120 | -0.0012 | 157.04935、133.02780、115.03851 |  |  |  |  |
| 18 | 3.88 | Catechin | C_15_H_14_O_6_ | 289.07178 | 289.07176 | 0.00002 | 271.00696、245.08163、179.05498 |  |  |  |  |
| 19 | 4.77 | Escinolide | C_9_H_6_O_4_ | 177.01819 | 177.01933 | -0.00114 | 161.04425、133.02835 |  |  |  |  |
| 20 | 4.84 | Chlorogenic acid | C_16_H_18_O_9_ | 353.08771 | 353.08781 | -0.0001 | 191.05508、173.04468、161.02345 |  |  |  |  |
| 21 | 5.13 | Caffeic acid | C_9_H_8_O_4_ | 179.03394 | 179.03498 | -0.00104 | 135.04362、107.04925 |  |  |  |  |
| 22 | 6.29 | Benzoic acid | C_7_H_6_O_2_ | 121.02792 | 121.02950 | -0.00158 | 93.03459、77.03967 |  |  |  |  |
| 23 | 7.89 | Vanillic acid | C_8_H_8_O_4_ | 167.03711 | 167.03498 | 0.00213 | 153.01799、123.04520、109.02951 |  |  |  |  |
| 24 | 8.91 | L-3-phenyllactic acid | C_9_H_10_O_3_ | 165.05443 | 165.05572 | -0.00129 | 148.04515、71.02793、 |  |  |  |  |
| 25 | 11.07 | Methyl chlorogenate | C_17_H_20_O_9_ | 367.10294 | 367.10346 | -0.00052 | 191.05502、179.03419、135.03912、93.03273 |  |  |  |  |
| 26 | 14.00 | Cinnamic acid | C_9_H_8_O_2_ | 147.04379 | 147.04515 | -0.00136 | 119.05017、102.94714、 |  |  |  |  |
| 27 | 14.26 | Vitexin-2-O-rhamnoside | C_27_H_30_O_14_ | 577.15558 | 577.15628 | -0.0007 | 413.08788、297.04053 |  |  |  |  |
| 28 | 15.23 | Rutin | C_27_H_30_O_16_ | 609.14551 | 609.14611 | -0.0006 | 301.03415、255.02963、151.00249 |  |  |  |  |
| 29 | 15.42 | Hyperoside | C_21_H_20_O_12_ | 463.08841 | 463.08820 | 0.00021 | 301.03421、271.02481、255.02928、178.99648、151.03871 |  |  |  |  |
| 30 | 16.24 | Vitexin | C_21_H_20_O_10_ | 431.09759 | 431.09837 | -0.00078 | 413.14517、297.04050 |  |  |  |  |
| 31 | 16.36 | Quercetin 3-O-arabinofuranoside | C_20_H_18_O_11_ | 433.07739 | 433.07763 | -0.00024 | 301.03290、271.02487、255.02959、 |  |  |  |  |
| 32 | 16.84 | Vicenin | C_27_H_30_O_15_ | 593.15070 | 593.15119 | -0.00049 | 575.14062、489.10381、459.09234、 |  |  |  |  |
| 33 | 16.88 | Naringenin-7-O-glucoside | C_21_H_22_O_10_ | 433.11414 | 433.11402 | 0.00012 | 271.06113、151.00230、107.01225 |  |  |  |  |
| 34 | 16.93 | Luteolin | C_15_H_10_O_6_ | 285.04053 | 285.04046 | 0.00007 | 243.02921、241.01460、217.05031、199.03885、175.03891、151.03851 |  |  |  |  |
| 35 | 16.96 | Quercitrin | C_21_H_20_O_11_ | 447.09308 | 447.09328 | -0.0002 | 301.20288、284.03262、255.02969、227.03432 |  |  |  |  |
| 36 | 17.71 | Quercetin | C_15_H_10_O_7_ | 301.03525 | 301.03538 | -0.00013 | 178.99745、151.00232、151.00232、107.01225、93.03290 |  |  |  |  |
| 37 | 18.07 | Naringenin | C_15_H_12_O_5_ | 271.06131 | 271.06120 | 0.00011 | 178.02155、151.00233、107.01227 |  |  |  |  |
| 38 | 18.16 | Kaempferol | C_15_H_10_O_6_ | 285.04047 | 285.04046 | 0.00001 | 257.04504、229.05028、185.05972、151.00232、133.02953 |  |  |  |  |
| 39 | 21.93 | Maslinic acid | C_30_H_48_O_4_ | 471.34912 | 471.34798 | 0.00114 | 453.34192、407.33151 |  |  |  |  |
| 40 | 24.01 | Linoleic acid | C_18_H_32_O_2_ |  |  |  |  | 281.24649 | 281.24751 | -0.00102 | 263.23584、221.22533、179.14233、165.12689 |
| 41 | 28.89 | 1. O-Acetyl Pomolic acid | C_32_H_50_O_5_ | 513.35931 | 513.35855 | 0.00076 | 495.35403、453.14035 |  |  |  |  |
| 42 | 29.06 | Betulinic acid | C_30_H_48_O_3_ |  |  |  |  | 457.36600 | 457.36762 | -0.00162 | 439.35556、411.32582、397.31013、 |
| 43 | 29.46 | Oleanolic acid | C_30_H_48_O_3_ |  |  |  |  | 457.36646 | 457.36762 | -0.00116 | 439.35583、411.36053、249.18434 |
| 44 | 31.80 | Betulin | C_30_H_50_O_2_ |  |  |  |  | 443.38983 | 443.38836 | 0.00147 | 425.37711、407.29532、221.19011 |
| 45 | 31.98 | Erythrodiol | C_30_H_50_O_2_ |  |  |  |  | 443.38681 | 443.38836 | -0.00155 | 425.37622、395.36612 |
| 46 | 33.34 | Palmitic acid | C_16_H_32_O_2_ | 255.23262 | 255.23295 | -0.00033 | 237.22162、211.24319、197.15478 |  |  |  |  |
| 47 | 34.14 | β-Amyrin | C_30_H_50_0 |  |  |  |  | 427.39383 | 427.39344 | 0.00039 | 409.38177、260.25812、191.17874 |
| 48 | 34.55 | Ursolic Acid | C_30_H_48_O_3_ | 455.35269 | 455.35307 | -0.00038 | 407.33246 |  |  |  |  |

**Supplement Table 2.** **Potential biomarkers in samples and corresponding metabolic pathways**

| Compound | RT  (min) | Mass | Formula | p | A/B | | C/B | | D/B | | E/B | | F/B | | KEGG | Pathway |
| --- | --- | --- | --- | --- | --- | --- | --- | --- | --- | --- | --- | --- | --- | --- | --- | --- |
|  |  |  |  |  | FC | Regulation | FC | Regulation | FC | Regulation | FC | Regulation | FC | Regulation |  |  |
| arachidonic acid | 12.95 | 304.2402 | C_20_H_32_O_2_ | 1.93E-06 | 1.4559 | down | 1.0472 | down | 1.0036 | down | 1.1374 | down | 1.0698 | down | C00219 | Arachidonic acid metabolism |
| Nicotinamide | 1.09 | 122.0483 | C_6_H_6_N_2_O | 4.31E-05 | 1.1447 | down | 1.2678 | up | 1.2782 | up | 1.1416 | up | 1.1667 | up | C00003 | Nicotinate and nicotinamide metabolism |
| valine | 9.27 | 116.0473 | C_5_H_8_O_3_ | 5.45E-05 | 1.0730 | down | 1.0503 | down | 1.0535 | down | 1.0885 | down | 1.1187 | down | C00141 | Valine, leucine and isoleucine degradation |
| Phenylacetylglycine | 4.45 | 193.0741 | C_10_H_11_NO_3_ | 2.11E-04 | 1.1832 | up | 1.1336 | down | 1.2980 | up | 1.6201 | up | 1.4031 | up | C05596 | Tyrosine metabolism |
| Indole | 3.28 | 117.058 | C_8_H_7_N | 7.59E-04 | 1.2253 | up | 1.0709 | up | 1.0811 | up | 1.3794 | up | 1.3375 | up | C00331 | Tryptophan metabolism |
| Skatole | 3.28 | 114.0471 | C_9_H_9_N | 0.0011 | 1.0417 | up | 1.1151 | down | 1.1191 | up | 1.2518 | up | 1.2058 | up | C08313 | Tryptophan metabolism |
| Acetyl-L-carnitine | 0.98 | 203.1158 | C_9_H_17_NO_4_ | 0.0012 | 1.0003 | down | 1.4265 | down | 1.4601 | down | 1.2186 | down | 1.1595 | down | C02571 | Insulin resistance |
| isoleucine | 9.9 | 131.0946 | C_6_H_13_NO_2_ | 0.0015 | 1.2583 | down | 1.1870 | down | 1.4022 | down | 1.6431 | down | 2.4129 | down | C00407 | Valine, leucine and isoleucine degradation |
| leucine | 9.04 | 131.0946 | C_6_H_13_NO_2_ | 0.0057 | 1.0100 | down | 1.0823 | down | 1.2297 | down | 1.1681 | down | 1.3281 | down | C00123 | Valine, leucine and isoleucine degradation |
| Panthenol | 3.22 | 205.1316 | C_9_H_19_NO_4_ | 0.0082 | 1.3417 | up | 1.1727 | up | 1.0081 | down | 1.3245 | up | 1.3485 | up | C05944 | Pantothenate and CoA biosynthesis |
| Tryptophan | 3.16 | 204.0899 | C_12_H_16_O | 0.0067 | 1.3145 | up | 1.1324 | up | 1.0178 | down | 1.3095 | up | 1.3647 | up | [C00078](https://www.kegg.jp/dbget-bin/www_bget?cpd:C00078) | Tryptophan metabolism |
| Kynurenic acid | 3.91 | 189.0428 | C_10_H_7_NO_3_ | 0.0099 | 1.3533 | up | 1.1806 | up | 1.0080 | down | 1.3186 | up | 1.3406 | up | C01717 | Tryptophan metabolism |
| 1-Pyrroline-4-hydroxy-2-carboxylate | 0.7440 | 129.0428 | C_12_H_10_O_2_ | 4.76E-05 | 1.0522 | down | 1.4399 | up | 1.1674 | up | 1.0270 | down | 1.0522 | down | [C04282](https://www.kegg.jp/dbget-bin/www_bget?cpd:C04282) | Arginine and proline metabolism |
| Palmitoleic acid | 12.9220 | 254.2244 | C_6_H_9_NO_3_ | 0.0029 | 4.5783 | down | 1.3212 | down | 1.1103 | up | 1.2953 | down | 1.4491 | up | [C08362](https://www.kegg.jp/dbget-bin/www_bget?cpd:C08362) | Fatty acid biosynthesis |
| Metanephrine | 2.2360 | 197.1054 | C_4_H_7_O_3_ | 0.0122 | 1.7398 | down | 1.3043 | up | 2.8889 | up | 1.3527 | down | 1.8592 | down | [C05588](https://www.kegg.jp/dbget-bin/www_bget?cpd:C05588) | Tyrosine metabolism |
